# Role of Biomarkers in Diagnosis and Prognostic Evaluation of Acute Pancreatitis

**DOI:** 10.1155/2015/519534

**Published:** 2015-08-05

**Authors:** Susanta Meher, Tushar Subhadarshan Mishra, Prakash Kumar Sasmal, Satyajit Rath, Rakesh Sharma, Bikram Rout, Manoj Kumar Sahu

**Affiliations:** Department of General Surgery, All India Institute of Medical Sciences, Bhubaneswar 751 019, India

## Abstract

Acute pancreatitis is a potentially life threatening disease. The spectrum of severity of the illness ranges from mild self-limiting disease to a highly fatal severe necrotizing pancreatitis. Despite intensive research and improved patient care, overall mortality still remains high, reaching up to 30–40% in cases with infected pancreatic necrosis. Although little is known about the exact pathogenesis, it has been widely accepted that premature activation of digestive enzymes within the pancreatic acinar cell is the trigger that leads to autodigestion of pancreatic tissue which is followed by infiltration and activation of leukocytes. Extensive research has been done over the past few decades regarding their role in diagnosis and prognostic evaluation of severe acute pancreatitis. Although many standalone biochemical markers have been studied for early assessment of severity, C-reactive protein still remains the most frequently used along with Interleukin-6. In this review we have discussed briefly the pathogenesis and the role of different biochemical markers in the diagnosis and severity evaluation in acute pancreatitis.

## 1. Introduction

Acute pancreatitis (AP) is a potentially life threatening disease with varying severity of presentation [1, 2]. Nearly 60%–80% of all cases of AP in developed countries are attributable to either gallstone disease or alcohol abuse [3, 4]. The incidence is similar in both sexes, although alcohol abuse is the more common cause in men and gallstones is the more common cause in women [5, 6]. There is an upsurge in the incidence of AP over the last few decades, although the case fatality rate has remained unchanged [7]. This may either be due to increased incidence of gallstone disease or improvement in diagnostic modalities [8].

The revised Atlanta classification system has classified AP into mild, moderate, and severe [9, 10]. More than 80% of acute pancreatitis attacks are mild and self-limiting and resolve without serious complications. In 20% of cases, however, it can be severe and complicated by major morbidity or mortality [3, 11, 12]. Moderate acute pancreatitis is characterized by the presence of transient organ failure or local/systemic complications [10]. Persistent organ failure is the feature of severe acute pancreatitis which is associated with a high rate of mortality. The overall mortality of AP is about 10–15% but reaches up to 30%–40% in patients with severe disease [13, 14]. Sepsis related multiorgan failure and infected pancreatic necrosis account for about 40–50% of all mortality in acute pancreatitis [13, 15, 16]. Mortality in AP occurs in two peaks [17–21]. Nearly 50% of deaths occur early within the first week due to massive inflammatory responses leading to multiorgan failure. Septic complications related to infected pancreatic necrosis leading to multiorgan failure are the prime cause of death, late in the disease [17–21]. The course and severity of AP can fluctuate rapidly and unpredictably [1, 22].

Despite the advances in investigational modalities and research techniques, the exact pathogenesis of AP is still unclear [18, 23–25]. Recent studies have suggested the role of inflammatory mediators and oxidative stress in the pathogenesis of AP and its sequelae [18, 23–25]. The pathophysiology of AP, role of various markers in establishing the diagnosis and prediction of severity, and upcoming markers including markers of oxidative stress are being discussed in this review.

## 2. Pathophysiology of Acute Pancreatitis

Despite intense research over centuries, the exact pathogenesis of AP remains elusive [3, 26]. Although many theories have been proposed, none of them appear to be complete [3, 27]. Some of the propositions include abnormal biliopancreatic duct common pathway theory, pancreatic autodigestion theory, gallstone migration theory, enzyme activation theory, kinin and complement activation theory, microcirculation disturbance theory, and pancreatic acinar cell apoptosis and necrosis theory, all of which are still controversial [3, 8]. They can only explain certain aspects of pathogenesis or suit disease due to specific aetiologies.

**Figure 1:**
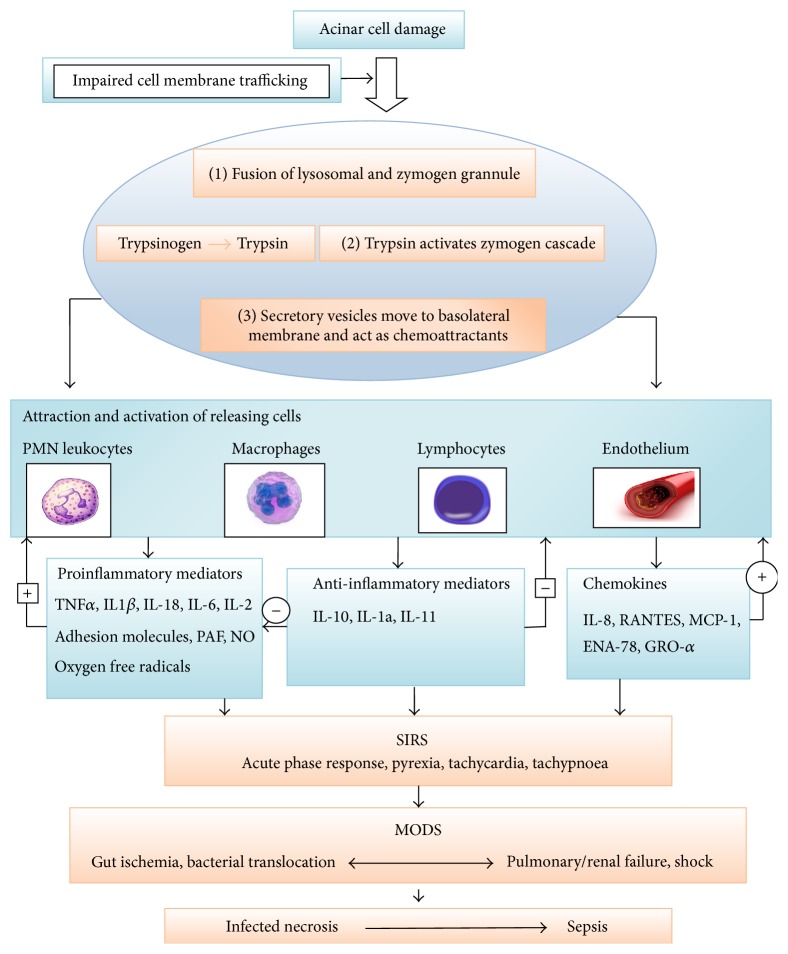
Schematic overview of pathogenesis of acute pancreatitis. Acinar cell damage leads to activation of trypsin following impairment of cell membrane trafficking with subsequent activation of zymogen cascade by trypsin. Attraction and activation of leukocyte occur with release of many proinflammatory and anti-inflammatory cytokines and also chemokines. An overt and sustained activation of proinflammatory mediators leads to Systemic Inflammatory Response Syndrome (SIRS) which may further proceed to multiorgan failure and infection of pancreatic necrosis and sepsis with late complications of acute pancreatitis [32, 33].

The biggest obstacle in the study of pathogenesis of AP is its rapid course and relative inaccessibility of pancreatic tissue [3]. To overcome this problem, investigators have now taken to animal models to study the molecular aspects of pathogenesis of acute pancreatitis [3, 28, 29]. Further complicating the issue are the paradoxical results about the pathogenesis, obtained from different animals exposed to similar aetiology [5]. The premature activation of trypsin in pancreatic parenchyma acting as the central step in the initiation of autodigestion of pancreatic tissue and subsequent local and systemic inflammation is presently the most accepted theory [17, 18, 30, 31]. Whatever is the initiating event, the disease progression can be viewed as a three-phase continuum: local inflammation of the pancreas and a generalized inflammatory response followed by the final stage of multiorgan dysfunction [17, 18, 30, 31].  Figure 1 illustrates the schematic overview of pathogenesis of acute pancreatitis [32, 33].

**Figure 2:**
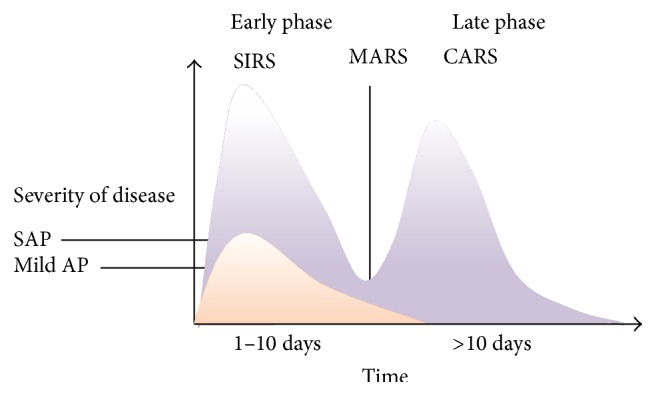
Two phases of severe acute pancreatitis (SAP). CARS: compensatory response syndrome; MARS: mixed antagonist response syndrome; SIRS: systemic inflammatory response syndrome; Mild AP: mild acute pancreatitis [1].

In the early phase, inflammation is usually localized to the pancreas which clinically manifests as mild acute pancreatitis. This usually resolves within a week without any local or systemic complications [5]. However, if the disease progresses, there occurs a phase of generalized inflammation, also known as systemic inflammatory response syndrome (SIRS) [1, 3]. Subsequently, there is a phase of mixed inflammatory response, known as mixed antagonist response syndrome (MARS), which clinically manifests as moderately severe acute pancreatitis, associated with transient organ failure and local complications [1]. Finally a phase of suppressed inflammatory response occurs which is known as compensatory response syndrome (CARS) which manifests as severe acute pancreatitis associated with persistent organ failure [1, 3]. The immune system in this phase is downregulated, leading to higher susceptibility of the pancreatic and peripancreatic tissue to infection from bacteria translocated from the gut. The ensuing sepsis and multiorgan failure are the major cause of late morbidity and mortality in severe acute pancreatitis [1, 33].  Figure 2 illustrates the two phases of severe acute pancreatitis.

## 3. Biomarkers in Establishing Diagnosis of AP

The diagnosis of acute pancreatitis is usually based on a combination of clinical findings, laboratory investigations, and imaging techniques. There is no gold standard test available to diagnose acute pancreatitis at present [34, 35]. According to revised Atlanta classification, diagnosis of acute pancreatitis requires two of the following three criteria [1, 22]:

1. Abdominal pain characteristic of AP (acute onset of a persistent, severe, epigastric pain often radiating to back).
2. Serum lipase (or amylase) activity at least three times greater than the upper limit of the reference interval.
3. Characteristic imaging findings of AP on contrast enhanced computed tomography (CECT) and less commonly magnetic resonance imaging (MRI) or transabdominal ultrasonography.

The pancreatic enzymes derived from pancreatic acinar cells [amylase, lipase, and the proenzyme trypsinogen] are the cornerstone in the laboratory diagnosis of AP [36]. Serum lipase is a more sensitive and specific biochemical marker of AP than the more frequently used amylase. Moreover, serum amylase level offers no additional advantage if simultaneously measured with serum lipase [36–38].

Additional biomarkers under evaluation for diagnosis of acute pancreatitis include pancreatic isoamylase, pancreatic elastase, serum trypsin, urinary trypsinogen activated peptide (TAP), Phospholipase A2, and Carboxypeptidase B (CAPB) [39, 40]. Serum trypsin and elastase are of particular interest because of their longer half-life which makes them useful in diagnosis during delayed presentations [41]. These tests, however, have not found much favor in clinical application because of a variety of reasons including inferior diagnostic accuracy compared to amylase and lipase, cumbersome techniques, or availability [33].

### 3.1. Amylase

Amylase is a glycoside hydrolase primarily produced in the pancreas and salivary glands and in very small quantities in other tissues. In acute pancreatitis, the blood level of amylase rapidly increases within six hours of onset of disease, exhibits a half-life of 10–12 hours, remains elevated for 3–5 days, and finally is excreted by the kidney [33, 36, 42]. After reaching a peak level, subsequent return of serum amylase to its normal level does not correlate with resolution of clinical symptoms [43]. Furthermore, the magnitude of the hyperamylasemia does not show significant statistical correlation with disease severity and ultimate prognosis [44]. In 19–32% of cases amylase activity may be normal at the time of hospital admission due to delayed presentation or exocrine pancreatic insufficiency (chronic alcoholism) [36, 43]. Raised serum amylase can also be found in many other intrabdominal inflammatory conditions and salivary disorders and in patients having decreased renal clearance. Macroamylasemia is a condition in which amylase remains bound to immunoglobulins or polysaccharides to form large molecular weight complexes leading to raised levels of serum amylase [36, 45, 46]. Hypertriglyceridemia competitively interferes with amylase assay, so a false low level of serum amylase can be found in patients having hypertriglyceridemia [36, 46]. Sensitivity and specificity of amylase as a diagnostic test for AP depend on its threshold value. At a cut-off level of 1000 IU/L, it has a sensitivity of around 55–84% and specificity up to 95% [36, 38, 46, 47].

### 3.2. Lipase

Lipase assay has a sensitivity and specificity of 80% and 60%, respectively [35, 48]. The serum concentration of lipase increases within 3–6 hours of onset of disease and peaks within 24 hours [32]. The increased serum level stays for around 7–14 days before it comes down to the normal level [32, 36]. In contrast to amylase, lipase is reabsorbed in renal tubules and stays for long at higher concentration, thereby giving greater sensitivity in patients with delayed presentation [32, 36]. Pancreatic lipase is four times more active than amylase and it is less affected by exocrine pancreatic deficiency occurring in patients of chronic pancreatitis [36, 49]. Hypertriglyceridemia does not influence the serum lipase assay as happens in the case of serum amylase. Patients taking frusemide can show increased lipase activity [36]. Increased serum level of lipase can also be seen in many intra-abdominal pathologies including acute cholecystitis, appendicitits, inflammatory bowel disease, intestinal ischemia, obstruction, perforation, and renal insufficiency [32, 36]. According to recent guidelines from UK, serum lipase should be preferred for diagnosis of AP over serum amylase wherever available [36–50]. At a cut-off level of 600 IU/L, most studies have reported specificity above 95%; however, serum lipase level's sensitivity is limited between 55–100% [36, 51]. Like that of amylase, most studies suggest a poor correlation between lipase activity and disease severity [44].

### 3.3. Trypsinogen

Trypsinogen is the zymogen of the pancreatic enzyme trypsin which is cleaved by duodenal enterokinase to produce the active enzyme trypsin and trypsinogen activated peptide (TAP) [22, 36]. Normally trypsinogen (trypsinogen-1 and trypsinogen-2) is secreted into the pancreatic fluid by the acinar cells, of which a small amount enters into the circulation and is excreted in urine. In pancreatitis large amounts of this enzyme enter the systemic circulation due to increased vascular permeability and there is a consequent increased clearance in urine. This forms the basis of the use of trypsinogen in the diagnosis and severity assessment of AP [32]. Both serum and urine concentrations rise within few hours of onset of disease and decline to normal level within 3 to 5 days [32, 36, 52]. A dipstick method using urinary trysinogen-2 has been devised for rapid detection of AP [32, 53]. Because of its low sensitivity and less availability, this test is less frequently used in routine clinical practice [32]. The greatest demerit of trypsinogen as a diagnostic test is its rapid clearance, which means it can only be used for early cases. It can be a useful test for screening of ERCP induced pancreatitis [32, 36].

## 4. Rationale of Severity Stratification and Its Assessment

Acute pancreatitis is self-limiting in 75%–80% of cases and does not require any treatment other than parenteral intravenous fluid, analgesics, and supportive care [4, 22, 23]. The remaining may suffer from severe attacks, with the mortality reaching up to 30%–50% [18]. This subgroup of patients needs to be identified early in the course of the disease and needs to be aggressively treated to prevent mortality. Proper identification of the mild disease is also necessary to avoid unnecessary over treatment, thereby reducing the financial implications.

## 5. Role of Biomarkers in Prediction of Severe Acute Pancreatitis

Severity assessment in acute pancreatitis was first started in 1974 by late Ranson et al. [54]. Since then a number of multifactorial scoring systems using common clinical and biochemical parameters have been described for prediction of severity. Ranson, Glasgow, and APACHE II score are few of the commonly used scoring systems [36]. Limitations of these scoring systems include delay in complete scoring where it takes 48 hours to complete Ranson and Glasgow scoring systems need a time of 48 hours to complete the assessment, while APACHE II score is very cumbersome to calculate [36]. The disadvantages of these prompt most of the researchers to find a single biochemical parameter which could accurately predict the severity of AP early in the course of the disease.

### 5.1. Interleukins

Interleukin-6 (IL-6) is produced by a wide range of cells like monocytes, macrophage, endothelium, and fibroblast in response to potent proinflammatory stimulus like TNF-alpha and IL-1*β* [33]. A large number of studies have already confirmed the role of IL-6 in early and accurate prediction of severity in acute pancreatitis [33, 36, 55]. Value of IL-6 is significantly elevated in SAP on the day of admission and tends to peak at 72 hrs after the clinical onset of disease, which makes IL-6 an excellent marker of early severity stratification [56]. In terms of predicting complications, IL-6 was found to be excellent in predicting remote organ failure, which is an integral part of severe acute pancreatitis [57]. Among various proinflammatory and anti-inflammatory cytokines, IL-6 has the best sensitivity and specificity for early assessment of SAP [58]. With a cut-off value of 50 pg/mL, Jiang et al. have found a sensitivity and specificity of 100% and 89.7%, respectively [59]. With a similar cut-off level, Khanna et al. found a sensitivity of 93.1% and specificity of 96.8% in their study [56]. The major drawback of IL-6 assay is that its serum concentration decreases very rapidly. Use of Il-6 in routine clinical practice is limited by its cost and the complexity of assay [36].

IL-8 is the best characterized member of the chemokine family studied in acute pancreatitis. It is a powerful secondary chemoattractant of neutrophil in the inflammatory process [60]. Many studies have shown promising results in early prediction of SAP [60]. One study has shown its role in monitoring life threatening complications in patients of necrotizing pancreatitis with multiorgan failure [61].

IL-12, IL-15, and IL-17 are proinflammatory cytokines which have been studied recently as potential biomarkers. Similar results have been seen in various studies as single biochemical markers on the day of admission. IL-15 and IL-17 are better predictors of organ dysfunction and mortality [62–64].

In a recent meta-analysis by Zhang et al., IL-6, IL-8, and IL-10 have shown promising results in predicting severe acute pancreatitis. They, however, found a lack of consensus regarding the ideal cut-off value for assessing the same [65].

### 5.2. C-Reactive Protein (CRP)

CRP is an acute phase reactant synthesized by the hepatocytes and is usually elevated in inflammatory conditions [66]. Cytokines like IL-6 are potent inducers of CRP synthesis in liver. It takes nearly 72 hours for the serum level of CRP to peak after the onset of symptoms [56]. It is the most frequently used single biomarker for assessment of severity in AP today. This is because it is inexpensive, widely available, and easy to measure [66]. A concentration of more than 150 mg/dL is often accepted as a predictor of severity in AP [56]. At this cut-off level, CRP has a sensitivity of 80–86% and specificity of 61–84% for diagnosing necrotizing pancreatitis within first 48 hours of onset of symptoms [56, 67]. In their study, Khanna et al. found a 100% sensitivity and 81.4% specificity for detection of pancreatic necrosis [56]. The demerit of CRP as marker is its delayed peak (48–72 hours) and its nonspecific nature as inflammatory marker. Before measurement of CRP, other inflammatory conditions such as cholangitis and pneumonia should be ruled out [56].

### 5.3. Procalcitonin (PCT)

It is a 116 amino acid propeptide of the hormone calcitonin which is released by hepatocytes and G-cells of the thyroid gland [56]. It is an acute phase reactant that has been extensively investigated as early marker in systemic bacterial infection, sepsis, and multiorgan failure [68]. Because severe acute pancreatitis is associated with sepsis, infected pancreatic necrosis, and multiorgan failure, procalcitonin can be used as a useful marker in early prediction of severity [69]. For faster result, PCT level can be measured by a semiquantitative strip test with a cut-off level of 0.5 ng/mL. For more accurate measurements however fully automated assay should be opted [70]. An increased PCT level has been found to be an early predictor of severity, pancreatic necrosis, and organ failure in patients with AP [70–72]. In a recent meta-analysis, a subgroup of 8 studies using PCT cut-off values of 0.5 ng/mL as discriminator found that the sensitivity and specificity of PCT for development of SAP were 73% and 87%, respectively, and overall area under curve (AUC) was 0.88. However, there was significant heterogeneity among individuals in the study [73]. In their study, Khanna et al. found 100% sensitivity of procalcitonin for prediction of organ failure and mortality, with a sensitivity of 86.4% for prediction of SAP [56]. Like that of Interleukin-6, procalcitonin assay is expensive and that is the reason why it is not used in routine clinical practice.

### 5.4. Polymorphonuclear Elastase (PMN Elastase)

PMN Elastase is the protease released by activated neutrophil as a first line defense following tissue injury [36]. Granulocyte infiltration and activation occur in the early phase of AP [74]. So PMN Elastase has been proved as an early marker of severe acute pancreatitis within 48 hours of onset of symptoms. With a cut-off level of 110 *µ*g/L, Domínguez-Muñoz et al. found a sensitivity and specificity of 92 and 91%, respectively, for detection of SAP within 48 hours of onset of symptoms. The positive and negative predictive values were 78% and 96%, respectively, and the area under the receiver operator curve was 0.956 [74]. Similar result has been found by Gross et al. and Wilson et al. in their study [75, 76]. More recent studies, however, by a Swiss group and the Japanese have yielded conflicting results [77, 78]. Domínguez-Muñoz et al. found quantification of plasma PMN elastase levels as a very accurate method for the early prognostic evaluation of AP and found its applicability in the clinical setting [74].

### 5.5. Tumor Necrosis Factor-Alpha (TNF-Alpha)

TNF-alpha is a macrophage derived pleotropic cytokine. It is thought to play major roles in pathophysiologic responses of inflammation following initial acinar cell injury. There are conflicting results among various studies regarding its role in prediction of severity in pancreatitis [79–81].

### 5.6. Markers for Trypsinogen Activation

#### 5.6.1. Trypsin-Alpha-1-Protease Inhibitor Complex

Many reports have shown its role in prediction of SAP. Its serum level is usually elevated early within 48 hours of the disease. It is, however, a nonspecific marker as its level can also be elevated in other gastrointestinal diseases like perforated ulcers [82–84].

#### 5.6.2. Trypsin Activation Peptide (TAP)

This is a small peptide released during the process of activation of trypsin from trypsinogen. TAP has been shown to be an excellent marker of severity in experimental models of AP. In humans, it is excreted in large amount in urine and peritoneal fluid. TAP activity increases early in the course of the disease and attains maximal value within 24–48 hours. Huang et al. did a meta-analysis on the role of urinary TAP in prediction of severity [85]. They found a sensitivity of 71% and specificity of 75% with an area under curve of 0.83 with a cut-off value of 35 nmol/L. This was comparable to the sensitivity and specificity of CRP and was better than that of APACHE II score. They found urinary TAP may be used as a potential severity stratification marker for acute pancreatitis [67, 85].

#### 5.6.3. Carboxypeptidase B Activation Peptide (CAPAP)

It is the largest activation peptide amongst the pancreatic proenzymes [86]. This peptide is very stable in urine and serum. In a study of 85 patients with acute pancreatitis CAPAP level correlated well, with an accuracy of 92%, in predicting development of pancreatic necrosis, whereas the level of its proenzyme did not show any correlation with pancreatic necrosis [87]. Both CAPAP and urinary TAP are excellent prognostic markers, although TAP is a better marker on the day of admission [88].

#### 5.6.4. Trysinogen-2

In acute pancreatitis the level of trysinogen-2 rises considerably more than that of trysinogen-1 [14]. High level of trypsinogen-2 can be found in both serum and urine. High serum level correlates better with complications and severity following ERCP induced pancreatitis [89–91]. High urinary trpsinogen-2 is used as a screening test for diagnosis of AP. A rapid dipstick method has been devised for rapid diagnosis of acute pancreatitis [92]. This test is particularly useful in rapid diagnosis of ERCP induced pancreatitis. Overall trysinogen-2 appears to be more useful as a diagnostic marker than as a predictor of severity [93].

## 6. Emerging Potential Biomarkers for Prediction of Severity in AP

### 6.1. Tissue Factor

Tissue factor is a transmembrane glycoprotein involved in the initiation of coagulation cascade. Recent studies have shown the usefulness of tissue factor as a marker for severity assessment. Andersson et al. in their study found that TF as a predictor of severity is not as good as IL-6 or CRP. High serum level early in the course may suggest a role in the pathogenesis of AP and give a window for therapeutic interventions [94].

### 6.2. Prealbumin to Fibrinogen Ratio

Prealbumin and Fibrinogen are acute phase reactants. Prealbumin is mostly used for assessment of nutritional status, whereas fibrinogen is used mostly for assessment of coagulation status in patients of acute pancreatitis. Ratio of prealbumin to fibrinogen has been studied recently as a severity marker in AP. According to Yue et al., it has superior sensitivity, specificity, positive predictive value (PPV), and NPV of 76.5%, 94.1%, 89.6%, and 85.6%, respectively, at a cut-off level of 31.70 mg/g than other scoring systems [95].

### 6.3. Cytokeratin 18

This is an epithelial cell structural protein, associated with apoptotic cell death. Recent animal studies have shown that wide apoptotic cell death is associated with a milder form of acute pancreatitis. High cytokeratin 18 level is found in patients with wide apoptotic cell death. Koruk et al. found a significantly high level of cytokeratin 18 in patients with mild acute pancreatitis (271.2 ± 45.5 versus 152.6 ± 38.2 IU/L; *p* < 0.001). M30 and M65 are newer ELISAs used to detect different circulating forms of cytokeratin 18 [96].

### 6.4. Hepcidin

Hepcidin is a protein which plays a key role in iron absorption in mammals. Abnormally high level of hepcidin can be found in acute inflammation. As it is primarily induced by IL-6, high level of hepcidin can be found in patients with acute pancreatitis. Based on this theory, Arabul et al. undertook a single centre prospective study to assess its role in prediction of severity in AP. They found hepcidin is a better predictive marker for SAP compared to CRP with an AUC of 0.79 versus 0.69, respectively [97].

### 6.5. Copeptin

Copeptin is a long amino acid peptide derived from a preprohormone consisting of neurophysin II, vasopressin, and copeptin. Its level rises during stress in critically ill patients. Isman et al. studied its role in acute pancreatitis as a predictive marker of severity. They found a significantly high concentration of copeptin at the time of admission in patients with SAP. Isman et al. also found that copeptin can be used as a novel prognostic marker for prediction of local complication, organ failure, and mortality in acute pancreatitis [98].

### 6.6. Soluble E-Selectin (sES) and Soluble Thrombomodulin (sTM)

Soluble ES is an endothelial activation marker, whereas soluble TM is an endothelial injury marker. During acute pancreatitis activated neutrophils release elastase which damages the endothelium. Ida et al. studied these two markers to find their significance in assessment of severe acute pancreatitis [99]. They concluded that those high levels of soluble ES can be found in all stages of the disease; therefore it can be used to monitor the disease severity. Soluble TM can be used as a predictive parker of mortality in acute pancreatitis on the first day of admission.

### 6.7. Endothelin 1

Elevated levels of endothelin have been found to be associated with acute pancreatitis with a strong correlation with the disease severity. High level of endothelin 1 can be used as a marker to monitor the disease progression [100].

### 6.8. Melatonin Concentration

Melatonin plays a protective role in the early phase of acute pancreatitis in the form of an antioxidant or scavengers of free radicals, inhibition of nuclear factor kappa B which indirectly prevents production of proinflammatory cytokines. It also modulates apoptosis and necrosis in acute pancreatitis. Variation in the level of melatonin can be used as a marker for prediction of SAP. Melatonin concentration below 28.74 ng/L has been found to be associated with severe acute pancreatitis as found by Jin et al. [101].

### 6.9. Serum Intercellular Adhesion Molecule-1 (ICAM-1)

Many previous reports have shown that ICAM-1 level increases significantly in acute pancreatitis. In a study of 36 patients, Zhu and Jiang found a sensitivity, specificity, positive predictive value, negative predictive value, positive likelihood ratio, and negative likelihood ratio of 61.11%, 71.4%, 0.6111, 0.7142, 2.1382, and 0.5445, respectively, at a cut-off level of 25 ng/mL [102]. The accuracy of detecting SAP was better than IL-6 and similar to APACHE II. It can be used as a reliable early marker within the first 24 hours for prediction of SAP in a rapid and simple manner.

### 6.10. Neutrophil Gelatinase-Associated Lipocalin (NGAL)

It is also known as human neutrophil lipocalin, lipocalin 2, and siderocalin. Lipocalin 2 is secreted by activated neutrophil in inflammation in which it binds with bacterial iron binding protein called siderophores, thus preventing bacterial infections by acting as bacteriostatic agent. Recently, studies have shown that this can be used as an early marker. Chakraborty et al. found a 100% sensitivity of detecting SAP within first 48 hours. It has also shown significant correlation with fatal complications and mortality in acute pancreatitis [103].

### 6.11. Total Calcium and Albumin Corrected Calcium

Total calcium and corrected calcium have shown similar efficacy like that of Ranson and APACHE II score in prediction of SAP. In a prospective study of 96 patients, Gutiérrez-Jiménez et al. have found sensitivity, specificity, positive predictive value, and negative predictive value of 67%, 82%, 27%, and 96% at a maximum cut-off level of 7.5 mg/dL for total calcium and 67%, 90%, 40%, and 96% for corrected calcium with a maximum cut-off level of 7.5 mg/dL [104].

### 6.12. Serum Proteomic Pattern

Serum proteomic profile has features which can differentiate mild from severe acute pancreatitis. This has been shown by Papachristou et al. who show 18 different signal intensities clusters out of 72 spectral clusters. Classification and regression tree (CART) analysis showed a primary splitter at 11,720 Da. After analysis it was found to have a sensitivity of 100% and specificity of 81% in discriminating mild from severe acute pancreatitis [105].

## 7. Biomarkers of Pancreatic Necrosis

Acute necrotizing pancreatitis is the deadliest form of AP with a very high mortality rate. Identification of pancreatic necrosis and infection early in the course of the disease is essential. A number of studies have been conducted over the last few decades to find a novel biomarker which can accurately predict pancreatic necrosis and infection in acute pancreatitis. However, there is a dearth of ideal and established biomarkers to indicate pancreatic necrosis (PN) in AP, an area now mired by controversies requiring extensive research. Following are few biomarkers with high positive predictive value in prediction of infected or sterile pancreatic necrosis.

### 7.1. Adipocytokines

Lipase mediated peripancreatic fat necrosis is associated with release of high levels of adipocytokines which can be used as marker for prediction of severity and pancreatic necrosis in AP. Adiponectin, resistin, leptin, and visfatin are the novel adipocytokines which have been studied recently as potential biomarkers in AP. In a comprehensive review of adipocytokines in nine human and three experimental studies, Karpavicius et al. found a significant correlation between high level of adipocytokines and SAP. Resistin and visfatin were found to be good predictors of pancreatic necrosis with cut-off levels of 11.9 ng/mL and 1.8 ng/mL, respectively. However, Al-Maramhy et al. did not find resistin as a useful marker for predicting severity [106, 107].

### 7.2. Matrix Metalloproteinase-9 (MMP-9)

MMP-9 is a Zn containing endopeptidase whose main function is extracellular matrix degradation. In the process of inflammation it is thought to be involved in neutrophil trafficking through the endothelial membrane. Recent studies on MMP-9 as potential biomarker in AP have shown a strong association of MMP-9 concentration at admission with subsequent development of pancreatic necrosis with a high sensitivity (91.7%) and positive predictive value (90.4%). It can also be used as a marker of disease severity and assessment of course of the disease [108, 109].

### 7.3. Macrophage Migration Inhibitory Factor (MIF)

It is a cytokine of the innate immunity system secreted from monocytes and macrophages. It is released in response to circulating lipopolysaccharides, gram positive exotoxins and proinflammatory cytokines. Rahman et al. observed that serum MIF concentrations were considerably elevated in patients of severe AP. This is typically seen in patients having PN involving more than 30% area of the pancreas as detected on contrast enhanced CT scan [110]. There was no correlation however between MIF levels and multiorgan failure in such patients. Macrophage Migration Inhibitory Factor is inexpensive and easily available. Efficacy of anti-MIF antibody has been proven in rodents by Calandra et al. and may act as target for future targeted therapy [111].

### 7.4. Fibrinogen-Like Protein-2 (fgl-2)

It is a new member of fibrinogen related protein superfamily, with direct prothrombinase and serine protease activity. Its activation results in fibrin deposition and microthrombosis lead to microvascular changes. High levels of fgl-2 closely correlate with the severity of AP and PN as a result of aforesaid mechanism in rats and may serve as a useful biomarker of severe AP in humans in times to come [112].

### 7.5. Cortisol Binding Globulin (CBG)

A recent study by Muller et al. has shown a significant difference in the peak level of CBG in the first 48 hours in patients having sterile (26.5 microg/mL) and infected pancreatic necrosis (16.0 microg/mL) at a cut-off level of 16.8 microg/mL. A decreased CBG level in the first 48 hours has been found as an early predictor of infected pancreatic necrosis in patients with AP with PPV and NPV of 100% and 87.5%, respectively [113].

### 7.6. Soluble Triggering Receptor Expressed on Myeloid Cells (sTERM1)

Lu et al. found sTERM1 as independent predictor of infected pancreatic necrosis at a cut-off level of 285.6 pg/mL (AUC: 0.972) in patients of AP [114].

### 7.7. IL-6 and PCT

These are established markers of infected pancreatic necrosis. PCT at a cut-off level of >2.0 ng mL is an independent predictor of infected pancreatic necrosis [115].

Many other studies including high serum creatinine level at admission, ghrelin, and nesfatin-1 did not reveal significant correlation as a predictive marker of pancreatic necrosis [116, 117].

## 8. Biomarkers of Organ Failure

### 8.1. Angiopoietin 2

Increased vascular permeability is the major cause of third space fluid loss which leads to organ failure in acute pancreatitis. Angiopoietin 1 and Angiopoietin 2 are modulators of vascular permeability which can be used as marker of persistent organ failure. Angiopoietin 2 has been recently evaluated as a marker of persistent organ failure in patients of severe acute pancreatitis. Whitcomb et al. did a multicentre prospective study to assess the role of angiopoietin 2 as an early marker of persistent organ failure in patients of SAP from USA and Germany [118]. They found that angiopoietin 2 level on the day of admission was significantly higher in patients with persistent organ failure with sensitivity, specificity, and area under curve of 90%, 67%, and 0.81, respectively. Buddingh et al. found a similar result in their randomized control trial. Angiopoietin 2 level was significantly higher in patients with SAP [6.4 versus 3.1 *µ*g/L (*p* < 0.001)]. In both studies angiopoietin 2 level was persistently high for initial 5–7 days which means that it can also be used to monitor the disease severity [119].

### 8.2. D-Dimer

Activation of coagulation cascade has been known to occur during the early phase of acute pancreatitis [120]. D-dimer of fibrinogen can be used as potential severity marker in AP. Studies have shown significantly different levels of D-dimer in patients of pancreatitis with or without complications [121]. In a recent study, Radenkovic et al. found D-dimer as novel marker for prediction of organ failure with a sensitivity of 90% and negative predictive value of 96% at a cut-off level of 414.00 microg/L [121]. Furthermore, it has been found that D-dimer level in pancreatitis correlates well with traditional markers like APACHE II and CRP levels [122]. According to Papachristou and Whitcomb D-dimer can be an easy, useful, and inexpensive early prognostic marker of SAP [123].

### 8.3. Soluble CD73

Many studies have shown that soluble CD73 can be used as a marker for early prediction of persistent organ failure. It has a low cost and it is simple to do but it is not as good as other parameters used for severity assessment [124].

## 9. Biomarkers of Oxidative Stress

Although pathogenesis of acute pancreatitis is not fully understood, there is evidence suggesting important role of oxidative stress in early stages of the disease as well as during disease progression. Sanfey et al. were the first to describe the involvement of oxygen free radicals in pathogenesis of acute pancreatitis [125]. Multiple clinical studies have shown higher oxidative stress levels in patients of acute pancreatitis than in healthy individuals. The level of oxidative stress marker increases with increase in disease severity [126–136]. Levels of antioxidants, lipid peroxidation products, and end products of action of reactive oxygen species (ROS) on biological molecules can help in assessing the oxidative stress of the patient with acute pancreatitis. After several studies, oxidative stress is now considered as key mediator of both local and systemic events occurring in acute pancreatitis [137, 138].

Animal studies with experimental acute pancreatitis have shown marked decrease in the levels of reduced glutathione in pancreas together with increase in lipid peroxidation products in the tissue and plasma. This suggests the presence of oxidative stress at tissue as well as systemic levels in acute pancreatitis [139]. Increased plasma levels of lipid peroxidation products, myeloperoxidase activity, and protein carbonyls are seen in patients with severe acute pancreatitis. These parameters correlate well with the severity of the disease in both clinical and experimental studies [131, 132, 140]. Thus increase in levels of malondialdehyde (MDA), one of the lipid peroxidation products, correlates directly with tissue injury and has also been associated with organ dysfunction in acute pancreatitis.

Multiple mechanisms are involved in triggering the expression of inflammatory genes and thus stimulating synthesis of proinflammatory molecules. Role of oxidative stress is complex and is yet to be clear. Antioxidant therapy in patients with acute pancreatitis has shown mixed results in human studies. Future studies are necessary to understand epigenetic modulation of proinflammatory genes in acute pancreatitis for better management of these patients.

## 10. Conclusion

Acute pancreatitis has been intensively studied worldwide. The overall mortality of the disease, however, has not improved significantly. Early aggressive management has been shown to reduce morbidity and mortality, for which early diagnosis and assessment of severity are essential. An ideal marker for early assessment of severity and predicting worsening of disease is lacking. Although IL-6 has shown promising result in assessment of the disease severity, its routine clinical use is limited by its cost and complexity of assay. C-reactive protein continues to be the most frequently used marker for severity assessment. Large population based multicentre studies of the available biomarkers need to be established which is ideal for predicting the disease severity and monitoring disease progression and which can be used routinely. Recently it has been found that oxidative stress plays an important role in the pathogenesis of AP. Further research into the biomarkers of oxidative stress and the role of antioxidants in limiting the disease progression will benefit the management of this otherwise unpredictable disease.

## References

[1] Phillip V., Steiner J. M., Algül H. (2014). Early phase of acute pancreatitis: assessment and management. *World Journal of Gastrointestinal Pathophysiology*.

[2] Nieminen A., Maksimow M., Mentula P. (2014). Circulating cytokines in predicting development of severe acute pancreatitis. *Critical Care*.

[3] Weitz G., Woitalla J., Wellhöner P., Schmidt K., Büning J., Fellermann K. (2015). Does etiology of acute pancreatitis matter? A review of 391 consecutive episodes. *Journal of the Pancreas*.

[4] Spanier B. W. M., Dijkgraaf M. G. W., Bruno M. J. (2008). Epidemiology, aetiology and outcome of acute and chronic pancreatitis: An update. *Best Practice and Research in Clinical Gastroenterology*.

[5] Lankisch P. G., Assmus C., Lehnick D., Maisonneuve P., Lowenfels A. B. (2001). Acute pancreatitis: does gender matter?. *Digestive Diseases and Sciences*.

[6] Dufour M. C., Adamson M. D. (2003). The epidemiology of alcohol induced pancreatitis. *Pancreas*.

[7] Goldacre M. J., Roberts S. E. (2004). Hospital admission for acute pancreatitis in an English population, 1963–98: database study of incidence and mortality. *British Medical Journal*.

[8] Yadav D., Lowenfels A. B. (2006). Trends in the epidemiology of the first attack of acute pancreatitis: a systematic review. *Pancreas*.

[9] Petrov M. S., Windsor J. A. (2010). Classification of the severity of acute pancreatitis: how many categories make sense?. *The American Journal of Gastroenterology*.

[10] Vege S. S., Gardner T. B., Chari S. T. (2009). Low mortality and high morbidity in severe acute pancreatitis without organ failure: A case for revising the Atlanta classification to include ‘moderately severe acute pancreatitis’. *American Journal of Gastroenterology*.

[11] Lund H., Tønnesen H., Tønnesen M. H., Olsen O. (2006). Long-term recurrence and death rates after acute pancreatitis. *Scandinavian Journal of Gastroenterology*.

[12] Swaroop V. S., Chari S. T., Clain J. E. (2004). Severe acute pancreatitis. *The Journal of the American Medical Association*.

[13] Dambrauskas Z., Giese N., Gulbinas A. (2010). Different profiles of cytokine expression during mild and severe acute pancreatitis. *World Journal of Gastroenterology*.

[14] Abu-Zidan F. M., Bonham M. J. D., Windsor J. A. (2000). Severity of acute pancreatitis: a multivariate analysis of oxidative stress markers and modified Glasgow criteria. *British Journal of Surgery*.

[15] Gloor B., Müller C. A., Worni M., Martignoni M. E., Uhl W., Büchler M. W. (2001). Late mortality in patients with severe acute pancreatitis. *British Journal of Surgery*.

[16] Uhl W., Warshaw A., Imrie C. (2002). IAP guidelines for the surgical management of acute pancreatitis. *Pancreatology*.

[17] Bhatia M., Brady M., Shokuhi S., Christmas S., Neoptolemos J. P., Slavin J. (2000). Inflammatory mediators in acute pancreatitis. *Journal of Pathology*.

[18] Bhatia M., Fei L. W., Cao Y. (2005). Pathophysiology of acute pancreatitis. *Pancreatology*.

[19] McKay C. J., Imrie C. W. (2004). The continuing challenge of early mortality in acute pancreatitis. *British Journal of Surgery*.

[20] Renner I. G., Savage W. T., Pantoja J. L., Renner V. J. (1985). Death due to acute pancreatitis. A retrospective analysis of 405 autopsy cases. *Digestive Diseases and Sciences*.

[21] Widdison A. L., Karanjia N. D. (1993). Pancreatic infection complicating acute pancreatitis. *British Journal of Surgery*.

[22] Banks P. A., Bollen T. L., Dervenis C. (2013). Classification of acute pancreatitis—2012: revision of the Atlanta classification and definitions by international consensus. *Gut*.

[23] Sandberg Å. A., Borgström A. (2002). Early prediction of severity in acute pancreatitis. Is this possible?. *Journal of the Pancreas*.

[24] Rinderknecht H. (1986). Activation of pancreatic zymogens. Normal activation, premature intrapancreatic activation, protective mechanisms against inappropriate activation. *Digestive Diseases and Sciences*.

[25] Glasbrenner B., Adler G. (1993). Pathophysiology of acute pancreatitis. *Hepato-Gastroenterology*.

[26] Pandol S. J. (2006). Acute pancreatitis. *Current Opinion in Gastroenterology*.

[27] Gabryelewicz A. (1995). Etiology and pathogenesis of acute pancreatitis—current view. *Roczniki Akademii Medycznej w Białymstoku*.

[28] Laukkarinen J. M., Weiss E. R., van Acker G. J. D., Steer M. L., Perides G. (2008). Protease-activated receptor-2 exerts contrasting model-specific effects on acute experimental pancreatitis. *The Journal of Biological Chemistry*.

[29] Samuel I., Tephly L., Williard D. E., Carter A. B. (2008). Enteral exclusion increases map kinase activation and cytokine production in a model of gallstone pancreatitis. *Pancreatology*.

[30] Bhatia M., Neoptolemos J. P., Slavin J. (2001). Inflammatory mediators as therapeutic targets in acute pancreatitis. *Current Opinion in Investigational Drugs*.

[31] M. Bhatia B. S. (2002). Novel therapeutic targets for acute pancreatitis and associated multiple organ dysfunction syndrome. *Current Drug Target—Inflammation & Allergy*.

[32] Lippi G., Valentino M., Cervellin G. (2012). Laboratory diagnosis of acute pancreatitis: in search of the Holy Grail. *Critical Reviews in Clinical Laboratory Sciences*.

[33] Rau B. M., Krüger C. M., Schilling M. K. (2005). Anti-cytokine strategies in acute pancreatitis: pathophysiological insights and clinical implications. *Roczniki Akademii Medycznej w Białymstoku*.

[34] Viljoen A., Patrick J. T. (2011). In search for a better marker of acute pancreatitis: third time lucky?. *Clinical Chemistry*.

[35] Chang J. W. Y., Chung C. H. (2011). Diagnosing acute pancreatitis: amylase or lipase?. *Hong Kong Journal of Emergency Medicine*.

[36] Matull W. R., Pereira S. P., O'Donohue J. W. (2006). Biochemical markers of acute pancreatitis. *Journal of Clinical Pathology*.

[37] Banks P. A., Freeman M. L. (2006). Practice guidelines in acute pancreatitis. *The American Journal of Gastroenterology*.

[38] Keim V., Teich N., Fiedler F., Hartig W., Thiele G., Mössner J. (1998). A comparison of lipase and amylase in the diagnosis of acute pancreatitis in patients with abdominal pain. *Pancreas*.

[39] Al-Bahrani A. Z., Ammori B. J. (2005). Clinical laboratory assessment of acute pancreatitis. *Clinica Chimica Acta*.

[40] Sigounas D. E., Tatsioni A., Christodoulou D. K., Tsianos E. V., Ioannidis J. P. A. (2011). New prognostic markers for outcome of acute pancreatitis: overview of reporting in 184 studies. *Pancreas*.

[41] Chase C. W., Barker D. E., Russell W. L., Phillip Burns R. (1996). Serum amylase and lipase in the evaluation of acute abdominal pain. *The American Surgeon*.

[42] Shah A. M., Eddi R., Kothari S. T., Maksoud C., DiGiacomo W. S., Baddoura W. (2010). Acute pancreatitis with normal serum lipase: a case series. *JOP*.

[43] Clavien P.-A., Robert J., Meyer P. (1989). Acute pancreatitis and normoamylasemia. Not an uncommon combination. *Annals of Surgery*.

[44] Vissers R. J., Abu-Laban R. B., McHugh D. F. (1999). Amylase and lipase in the emergency department evaluation of acute pancreatitis. *Journal of Emergency Medicine*.

[45] Spechler S. J., Dalton J. W., Robbins A. H. (1983). Prevalence of normal serum amylase levels in patients with acute alcoholic pancreatitis. *Digestive Diseases and Sciences*.

[46] Yadav D., Agarwal N., Pitchumoni C. S. (2002). A critical evaluation of laboratory tests in acute pancreatitis. *The American Journal of Gastroenterology*.

[47] Smith R. C., Southwell-Keely J., Chesher D. (2005). Should serum pancreatic lipase replace serum amylase as a biomarker of acute pancreatitis?. *ANZ Journal of Surgery*.

[48] Lott J. A., Patel S. T., Sawhney A. K., Kazmierczak S. C., Love J. E. (1986). Assays of serum lipase: analytical and clinical considerations. *Clinical Chemistry*.

[49] Tietz N. W., Shuey D. F. (1993). Lipase in serum: the elusive enzyme: an overview. *Clinical Chemistry*.

[50] Werner J., Feuerbach S., Uhl W., Büchler M. W. (2005). Management of acute pancreatitis: from surgery to interventional intensive care. *Gut*.

[51] Kylänpää-Bäck M.-L., Kemppainen E., Puolakkainen P. (2002). Comparison of urine trypsinogen-2 test strip with serum lipase in the diagnosis of acute pancreatitis. *Hepato-Gastroenterology*.

[52] Petersson U., Appelros S., Borgström A. (1999). Different patterns in immunoreactive anionic and cationic trypsinogen in urine and serum in human acute pancreatitis. *International Journal of Pancreatology*.

[53] Kamer E., Unalp H. R., Derici H., Tansug T., Onal M. A. (2007). Early diagnosis and prediction of severity in acute pancreatitis using the urine trypsinogen-2 dipstick test: a prospective study. *World Journal of Gastroenterology*.

[54] Ranson J. H., Rifkind K. M., Roses D. F., Fink S. D., Eng K., Spencer F. C. (1974). Prognostic signs and the role of operative management in acute pancreatitis. *Surgery Gynecology and Obstetrics*.

[55] Heath D. I., Cruickshank A., Gudgeon M., Jehanli A., Shenkin A., Imrie C. W. (1993). Role of interleukin-6 in mediating the acute phase protein response and potential as an early means of severity assessment in acute pancreatitis. *Gut*.

[56] Khanna A. K., Meher S., Prakash S. (2013). Comparison of Ranson, Glasgow, MOSS, SIRS, BISAP, APACHE-II, CTSI Scores, IL-6, CRP, and procalcitonin in predicting severity, organ failure, pancreatic necrosis, and mortality in acute pancreatitis. *HPB Surgery*.

[57] De Beaux A. C., Goldie A. S., Ross J. A., Carter D. C., Fearon K. C. H. (1996). Serum concentrations of inflammatory mediators related to organ failure in patients with acute pancreatitis. *British Journal of Surgery*.

[58] Rettally C. A., Skarda S., Garza M. A., Schenker S. (2003). The usefulness of laboratory tests in the early assessment of severity of acute pancreatitis. *Critical Reviews in Clinical Laboratory Sciences*.

[59] Jiang C.-F., Shiau Y.-C., Ng K.-W., Tan S.-W. (2004). Serum interleukin-6, tumor necrosis factor alpha and C-reactive protein in early prediction of severity of acute pancreatitis. *Journal of the Chinese Medical Association*.

[60] Gross V., Andreesen R., Leser H.-G. (1992). Interleukin-8 and neutrophil activation in acute pancreatitis. *European Journal of Clinical Investigation*.

[61] Rau B., Steinbach G., Gansauge F., Mayer J. M., Grünert A., Beger H. G. (1997). The role of interleukin-8 in the severity assessment of septic complications in necrotizing pancreatitis. *Digestion*.

[62] Gregorić P., Doklestić K., Stanković S. (2014). Interleukin-12 as a predictor of outcome in patients with severe acute pancreatitis. *Hepato-Gastroenterology*.

[63] Ueda T., Takeyama Y., Yasuda T. (2007). Serum interleukin-15 level is a useful predictor of the complications and mortality in severe acute pancreatitis. *Surgery*.

[64] Botoi G., Andercou A. (2009). Interleukin 17–prognostic marker of severe acute pancreatitis. *Chirurgia*.

[65] Zhang J., Niu J., Yang J. (2014). Interleukin-6, interleukin-8 and interleukin-10 in estimating the severity of acute pancreatitis: an updated meta-analysis. *Hepato-Gastroenterology*.

[66] Wilson C., Heads A., Shenkin A., Imrie C. W. (1989). C-reactive protein, antiproteases and complement factors as objective markers of severity in acute pancreatitis. *British Journal of Surgery*.

[67] Neoptolemos J. P., Kemppainen E. A., Mayer J. M. (2000). Early prediction of severity in acute pancreatitis by urinary trypsinogen activation peptide: a multicentre study. *The Lancet*.

[68] Al-Nawas B., Krammer I., Shah P. M. (1996). Procalcitonin in diagnosis of severe infections. *European Journal of Medical Research*.

[69] Woo S. M., Noh M. H., Kim B. G. (2011). Comparison of serum procalcitonin with Ranson, APACHE-II, Glasgow and Balthazar CT severity index scores in predicting severity of acute pancreatitis. *The Korean Journal of Gastroenterology*.

[70] Kylänpää-Bäck M.-L., Takala A., Kemppainen E., Puolakkainen P., Haapiainen R., Repo H. (2001). Procalcitonin strip test in the early detection of severe acute pancreatitis. *British Journal of Surgery*.

[71] Mandi Y., Farkas G., Takacs T., Boda K., Lonovics J. (2000). Diagnostic relevance of procalcitonin, IL-6, and sICAM-1 in the prediction of infected necrosis in acute pancreatitis. *International Journal of Pancreatology*.

[72] Bülbüller N., Doğru O., Ayten R., Akbulut H., Ilhan Y. S., Çetinkaya Z. (2006). Procalcitonin is a predictive marker for severe acute pancreatitis. *Ulusal Travma ve Acil Cerrahi Dergisi*.

[73] Mofidi R., Suttie S. A., Patil P. V., Ogston S., Parks R. W. (2009). The value of procalcitonin at predicting the severity of acute pancreatitis and development of infected pancreatic necrosis: systematic review. *Surgery*.

[74] Domínguez-Muñoz J. E., Villanueva A., Lariño J. (2006). Accuracy of plasma levels of polymorphonuclear elastase as early prognostic marker of acute pancreatitis in routine clinical conditions. *European Journal of Gastroenterology & Hepatology*.

[75] Gross V., Schölmerich J., Leser H. G. (1990). Granulocyte elastase in assessment of acute pancreatitis. *Digestive Diseases and Sciences*.

[76] Wilson R. B., Warusavitarne J., Crameri D. M., Alvaro F., Davies D. J., Merrett N. (2005). Serum elastase in the diagnosis of acute pancreatitis: a prospective study. *ANZ Journal of Surgery*.

[77] Robert J. H., Frossard J. L., Mermillod B. (2002). Early prediction of acute pancreatitis: prospective study comparing computed tomography scans, Ranson, Glasgow, Acute Physiology and Chronic Health Evaluation II scores, and various serum markers. *World Journal of Surgery*.

[78] Mayer J. M., Raraty M., Slavin J. (2002). Serum amyloid A is a better early predictor of severity than C-reactive protein in acute pancreatitis. *British Journal of Surgery*.

[79] Exley A. R., Leese T., Holliday M. P., Swann R. A., Cohen J. (1992). Endotoxaemia and serum tumour necrosis factor as prognostic markers in severe acute pancreatitis. *Gut*.

[80] McKay C. J., Gallagher G., Brooks B., Imrie C. W., Baxter J. N. (1996). Increased monocyte cytokine production in association with systemic complications in acute pancreatitis. *British Journal of Surgery*.

[81] Domínguez-Muñoz J. E., Carballo F., García M. J. (1993). Monitoring of serum proteinase-antiproteinase balance and systemic inflammatory response in prognostic evaluation of acute pancreatitis. Results of a prospective multicenter study. *Digestive Diseases and Sciences*.

[82] Borgström A., Lasson Å. (1984). Trypsin-alpha 1-protease inhibitor complexes in serum and clinical course of acute pancreatitis. *Scandinavian Journal of Gastroenterology*.

[83] Hedström J., Sainio V., Kemppainen E. (1996). Serum complex of trypsin 2 and alpha 1 antitrypsin as diagnostic and prognostic marker of acute pancreatitis: clinical study in consecutive patients. *British Medical Journal*.

[84] Sultan S., Baillie J. (2002). What are the predictors of post-ERCP pancreatitis, and how useful are they?. *Journal of the Pancreas*.

[85] Huang W., Altaf K., Jin T. (2013). Prediction of the severity of acute pancreatitis on admission by urinary trypsinogen activation peptide: a meta-analysis. *World Journal of Gastroenterology*.

[86] Appelros S., Thim L., Borgström A. (1998). The activation peptide of carboxypeptidase B in serum and urine in acute pancreatitis. *Gut*.

[87] Müller C. A., Appelros S., Uhl W., Büchler M. W., Borgström A. (2002). Serum levels of procarboxypeptidase B and its activation peptide in patients with acute pancreatitis and non-pancreatic diseases. *Gut*.

[88] Sáez J., Martínez J., Trigo C. (2002). Comparative study of the activation peptide of carboxypeptidase B and of trypsinogen as early predictors of the severity of acute pancreatitis. *Pancreatology*.

[89] Hedström J., Kemppainen E., Andersén J., Jokela H., Puolakkainen P., Stenman U.-H. (2001). A comparison of serum trypsinogen-2 and trypsin-2-alpha1-antitrypsin complex with lipase and amylase in the diagnosis and assessment of severity in the early phase of acute pancreatitis. *American Journal of Gastroenterology*.

[90] Lempinen M., Kylänpää-bäck M.-L., Stenman U.-H. (2001). Predicting the severity of acute pancreatitis by rapid measurement of trypsinogen-2 in urine. *Clinical Chemistry*.

[91] Kemppainen E., Hedström J., Puolakkainen P. (1997). Increased serum trypsinogen 2 and trypsin 2-alpha-1-antitrypsin complex values identify endoscopic retrograde cholangiopancreatography induced pancreatitis with high accuracy. *Gut*.

[92] Hedström J., Korvuo A., Kenkimäki P. (1996). Urinary trypsinogen-2 test strip for acute pancreatitis. *The Lancet*.

[93] Johnson C. D., Lempinen M., Imrie C. W. (2004). Urinary trypsinogen activation peptide as a marker of severe acute pancreatitis. *British Journal of Surgery*.

[94] Andersson E., Axelsson J., Eckerwall G., Ansari D., Andersson R. (2010). Tissue factor in predicted severe acute pancreatitis. *World Journal of Gastroenterology*.

[95] Yue W., Liu Y., Ding W. (2015). The predictive value of the prealbumin-to-fibrinogen ratio in patients with acute pancreatitis. *International Journal of Clinical Practice*.

[96] Koruk İ., Özdemir H., Aydinli M., Tarakçioğlu M., Koruk M. (2012). The relation between serum cytokeratin 18 and acute pancreatitis: can it be a serological predictive marker?. *Turkish Journal of Gastroenterology*.

[97] Arabul M., Celik M., Aslan O. (2013). Hepcidin as a predictor of disease severity in acute pancreatitis: a single center prospective study. *Hepato-Gastroenterology*.

[98] Isman F. K., Zulfikaroglu B., Isbilen B. (2013). Copeptin is a predictive biomarker of severity in acute pancreatitis. *The American Journal of Emergency Medicine*.

[99] Ida S., Fujimura Y., Hirota M. (2009). Significance of endothelial molecular markers in the evaluation of the severity of acute pancreatitis. *Surgery Today*.

[100] Bennett J., Cooper D., Balakrishnan A., Rhodes M., Lewis M. (2006). Is there a role for serum endothelin in predicting the severity of acute pancreatitis?. *Hepatobiliary and Pancreatic Diseases International*.

[101] Jin Y., Lin C.-J., Dong L.-M., Chen M.-J., Zhou Q., Wu J.-S. (2013). Clinical significance of melatonin concentrations in predicting the severity of acute pancreatitis. *World Journal of Gastroenterology*.

[102] Zhu H.-H., Jiang L.-L. (2012). Serum inter-cellular adhesion molecule 1 is an early marker of diagnosis and prediction of severe acute pancreatitis. *World Journal of Gastroenterology*.

[103] Chakraborty S., Kaur S., Muddana V. (2010). Elevated serum neutrophil gelatinase-associated lipocalin is an early predictor of severity and outcome in acute pancreatitis. *American Journal of Gastroenterology*.

[104] Gutiérrez-Jiménez A. A., Castro-Jiménez E., Lagunes-Córdoba R. (2014). Total serum calcium and corrected calcium as severity predictors in acute pancreatitis. *Revista de Gastroenterologia de Mexico*.

[105] Papachristou G. I., Malehorn D. E., Lamb J., Slivka A., Bigbee W. L., Whitcomb D. C. (2007). Serum proteomic patterns as a predictor of severity in acute pancreatitis. *Pancreatology*.

[106] Karpavicius A., Dambrauskas Z., Sileikis A., Vitkus D., Strupas K. (2012). Value of adipokines in predicting the severity of acute pancreatitis: comprehensive review. *World Journal of Gastroenterology*.

[107] Al-Maramhy H., Abdelrahman A. I., Sawalhi S. (2014). Resistin is not an appropriate biochemical marker to predict severity of acute pancreatitis: a case-controlled study. *World Journal of Gastroenterology*.

[108] Chen P., Yuan Y., Wang S., Zhan L., Xu J. (2006). Serum matrix metalloproteinase-9 as a marker for the assessment of severe acute pancreatitis. *Tohoku Journal of Experimental Medicine*.

[109] Guo J., Xue P., Yang X.-N., Liu X.-B., Huang W., Xia Q. (2012). Serum matrix metalloproteinase-9 is an early marker of pancreatic necrosis in patients with severe acute pancreatitis. *Hepato-Gastroenterology*.

[110] Rahman S. H., Menon K. V., Holmfield J. H. M., McMahon M. J., Guillou J. P. (2007). Serum macrophage migration inhibitory factor is an early marker of pancreatic necrosis in acute pancreatitis. *Annals of Surgery*.

[111] Calandra T., Froidevaux C., Martin C., Roger T. (2003). Macrophage migration inhibitory factor and host innate immune defenses against bacterial sepsis. *Journal of Infectious Diseases*.

[112] Ye X.-H., Chen T.-Z., Huai J.-P. (2013). Correlation of fibrinogen-like protein 2 with progression of acute pancreatitis in rats. *World Journal of Gastroenterology*.

[113] Muller C. A., Belyaev O., Vogeser M. (2007). Corticosteroid-binding globulin: a possible early predictor of infection in acute necrotizing pancreatitis. *Scandinavian Journal of Gastroenterology*.

[114] Lu Z., Liu Y., Dong Y.-H. (2012). Soluble triggering receptor expressed on myeloid cells in severe acute pancreatitis: a biological marker of infected necrosis. *Intensive Care Medicine*.

[115] Müller C. A., Uhl W., Printzen G. (2000). Role of procalcitonin and granulocyte colony stimulating factor in the early prediction of infected necrosis in severe acute pancreatitis. *Gut*.

[116] Türkoğlu A., Böyük A., Tanriverdi M. H. (2014). The potential role of BMI, plasma leptin, nesfatin-1 and ghrelin levels in the early detection of pancreatic necrosis and severe acute pancreatitis: a prospective cohort study. *International Journal of Surgery*.

[117] Muddana V., Whitcomb D. C., Khalid A., Slivka A., Papachristou G. I. (2009). Elevated serum creatinine as a marker of pancreatic necrosis in acute pancreatitis. *American Journal of Gastroenterology*.

[118] Whitcomb D. C., Muddana V., Langmead C. J. (2010). Angiopoietin-2, a regulator of vascular permeability in inflammation, is associated with persistent organ failure in patients with acute pancreatitis from the United States and Germany. *The American Journal of Gastroenterology*.

[119] Buddingh K. T., Koudstaal L. G., Van Santvoort H. C. (2014). Early angiopoietin-2 levels after onset predict the advent of severe pancreatitis, multiple organ failure, and infectious complications in patients with acute pancreatitis. *Journal of the American College of Surgeons*.

[120] Ke L., Ni H. B., Tong Z. H., Li W. Q., Li N., Li J. S. (2012). d-dimer as a marker of severity in patients with severe acute pancreatitis. *Journal of Hepato-Biliary-Pancreatic Sciences*.

[121] Radenkovic D., Bajec D., Ivancevic N. (2009). D-dimer in acute pancreatitis: a new approach for an early assessment of organ failure. *Pancreas*.

[122] Boskovic A., Pasic S., Soldatovic I., Milinic N., Stankovic I. (2014). The role of D-dimer in prediction of the course and outcome in pediatric acute pancreatitis. *Pancreatology*.

[123] Papachristou G. I., Whitcomb D. C. (2004). Predictors of severity and necrosis in acute pancreatitis. *Gastroenterology Clinics of North America*.

[124] Maksimow M., Kyhälä L., Nieminen A. (2014). Early prediction of persistent organ failure by soluble CD73 in patients with acute pancreatitis^∗^. *Critical Care Medicine*.

[125] Sanfey H., Bulkley G. B., Cameron J. L. (1984). The role of oxygen-derived free radicals in the pathogenesis of acute pancreatitis. *Annals of Surgery*.

[126] Scott P., Bruce C., Schofield D., Shiel N., Braganza J. M., McCloy R. F. (1993). Vitamin C status in patients with acute pancreatitis. *British Journal of Surgery*.

[127] Braganza J. M., Scott P., Bilton D. (1995). Evidence of early oxidative stress in acute pancreatitis. Clues for correction. *International Journal of Pancreatology*.

[128] Tsai K., Wang S.-S., Chen T.-S. (1998). Oxidative stress: an important phenomenon with pathogenetic significance in the progression of acute pancreatitis. *Gut*.

[129] Wereszczyńska-Siemia̧tkowska U., Da̧browski A., Jedynak M., Gabryelewicz A. (1998). Oxidative stress as an early prognostic factor in acute pancreatitis (AP): its correlation with serum phospholipase A2 (PLA2) and plasma polymorphonuclear elastase (PMN-E) in different-severity forms of human AP. *Pancreas*.

[130] Curran F. J. M., Sattar N., Talwar D., Baxter J. N., Imrie C. W. (2000). Relationship of carotenoid and vitamins A and E with the acute inflammatory response in acute pancreatitis. *British Journal of Surgery*.

[131] Park B. K., Chung J. B., Lee J. H. (2003). Role of oxygen free radicals in patients with acute pancreatitis. *World Journal of Gastroenterology*.

[132] Winterbourn C. C., Bonham M. J. D., Buss H., Abu-Zidan F. M., Windsor J. A. (2003). Elevated protein carbonyls as plasma markers of oxidative stress in acute pancreatitis. *Pancreatology*.

[133] Wereszczynska-Siemiatkowska U., Mroczko B., Siemiatkowski A., Szmitkowski M., Borawska M., Kosel J. (2004). The importance of interleukin 18, glutathione peroxidase, and selenium concentration changes in acute pancreatitis. *Digestive Diseases and Sciences*.

[134] Rahman S. H., Ibrahim K., Larvin M., Kingsnorth A., McMahon M. J. (2004). Association of antioxidant enzyme gene polymorphisms and glutathione status with severe acute pancreatitis. *Gastroenterology*.

[135] Dziurkowska-Marek A., Marek T. A., Nowak A., Kacperek-Hartleb T., Sierka E., Nowakowska-Duława E. (2004). The dynamics of the oxidant-antioxidant balance in the early phase of human acute biliary pancreatitis. *Pancreatology*.

[136] Thareja S., Bhardwaj P., Sateesh J., Saraya A. (2009). Variations in the levels of oxidative stress and antioxidants during early acute pancreatitis. *Tropical Gastroenterology*.

[137] Escobar J., Pereda J., Arduini A. (2012). Oxidative and nitrosative stress in acute pancreatitis. Modulation by pentoxifylline and oxypurinol. *Biochemical Pharmacology*.

[138] Que R.-S., Cao L.-P., Ding G.-P., Hu J.-A., Mao K.-J., Wang G.-F. (2010). Correlation of nitric oxide and other free radicals with the severity of acute pancreatitis and complicated systemic inflammatory response syndrome. *Pancreas*.

[139] Rau B., Poch B., Gansauge F. (2000). Pathophysiologic role of oxygen free radicals in acute pancreatitis: initiating event or mediator of tissue damage?. *Annals of Surgery*.

[140] Hernández V., Miranda M., Pascual I. (2011). Malondialdehyde in early phase of acute pancreatitis. *Revista Espanola de Enfermedades Digestivas*.

